# Couple communication and contraception use in urban Senegal

**DOI:** 10.1177/20503121211023378

**Published:** 2021-06-04

**Authors:** Brigid K Grabert, Ilene S Speizer, Marisa Elena Domino, Leah Frerichs, Amy Corneli, Bruce J Fried

**Affiliations:** 1 Lineberger Comprehensive Cancer Center, The University of North Carolina at Chapel Hill, Chapel Hill, NC, USA; 2 Department of Health Behavior, UNC Gillings School of Global Public Health, Chapel Hill, NC, USA; 3 Department of Maternal and Child Health, UNC Gillings School of Global Public Health, Chapel Hill, NC, USA; 4 Carolina Population Center, The University of North Carolina at Chapel Hill, Chapel Hill, NC, USA; 5 Department of Health Policy and Management, UNC Gillings School of Global Public Health, Chapel Hill, NC, USA; 6 Cecil G. Sheps Center for Health Services Research, The University of North Carolina at Chapel Hill, Chapel Hill, NC, USA; 7 Department of Population Health Sciences, Duke University School of Medicine, Durham, NC, USA

**Keywords:** Couple communication, contraception, Senegal, polygyny, epidemiology/public health, women’s health

## Abstract

**Objectives:**

Couple communication about family planning has been shown to increase uptake of contraception. However, couple communication is often measured based solely on one partner’s report of communication. This research investigates the influence of couple-reported communication about family planning on current and future use of contraception using couple-level data.

**Methods:**

We used baseline data from the Measurement, Learning, and Evaluation (MLE) project collected through household surveys in 2011 from a cross-sectional representative sample of women and men in urban Senegal to conduct secondary data analysis. We used multivariable logit models to estimate the average marginal effects of couple communication about family planning on current contraception use and future intention to use contraception.

**Results:**

Couple communication about family planning reported by both partners was significantly associated with an increased likelihood of current use of contraception and with future intention to use contraception among non-contracepting couples. Couples where one partner reported discussing family planning had a 25% point greater likelihood of current contraception use than couples where neither partner reported discussing, while couples where both partners reported discussing family planning had a 56% point greater likelihood of current contraception use, representing more than twice the effect size. Among couples not using contraception, couples where one partner reported discussing family planning had a 15% point greater likelihood of future intention to use contraception than couples where neither partner reported discussing, while couples where both partners reported discussing family planning had a 38% point greater likelihood of future intention to use contraception.

**Conclusion:**

These findings underscore the importance of the inclusion of both partners in family planning programs to increase communication about contraception and highlight the need for future research using couple-level data, measures, and analysis.

## Introduction

Western sub-Saharan Africa has some of the world’s highest fertility, and maternal and infant mortality rates.^1–3^ In Senegal, the maternal mortality ratio was 315 deaths per 100,000 live births^4^ and the neonatal mortality rate was approximately 21 deaths per 1000 live births in 2017.^5^ These numbers are much higher than the international Sustainable Development Goal targets of fewer than 70 maternal deaths per 100,000 live births and fewer than 12 neonatal deaths per 1000 live births by 2030.^6^ The total fertility rate (TFR) in Senegal was 4.6 births per woman in 2018, compared to a global TFR of 2.4.^7,8^ The unmet need for contraception in Senegal in 2017 was 22%, and the contraceptive prevalence rate was 27% for women in union.^9^ Increasing contraceptive use among fecund women who want to limit or space births according to their preferences (i.e. reducing unmet need)^10^ lowers the risk of both maternal and infant mortality.^11–13^

Research has demonstrated the effects of numerous individual-level factors on contraceptive use, including the educational level of women and men, women’s employment, the number of previous births, and attitudes toward contraception.^12,14–17^ Most of the studies demonstrating the effect of these factors focus exclusively on women (or men) and not on couples or on other higher-level contextual factors, such as regional differences. However, reproductive decision-making does not occur solely at the individual level.^18^ A recent review found that there is limited research about the influence of the greater social context on reproductive health decisions, beyond individual-level factors, and specifically noted the lack of research examining how men and women make reproductive health decisions as a couple.^19^ For example, some research has shown that opposition from others, including spouses, mothers-in-law, or providers and community members may influence women’s, men’s, and couple’s family planning decisions.^17,20–24^ A couple-level analysis from Ethiopia found that husband’s opinions about contraception and childbearing carried more weight than the opinions of his female partner.^25^ In the 2018 Senegal Demographic and Health Survey, men in union reported a higher ideal number of children (8.0) on average than women in union (5.9),^26^ and a husband’s higher ideal number of children may influence his wife’s decisions to use (or not use) contraception.^20^ Social norms may also limit contraceptive use: in a recent study in Senegal, 25% of women believed that their religion (primarily Islam) prohibited family planning use.^20^

Many studies have concluded that couple communication influences family planning decision-making,^25,27–33^ but previous research about couple communication and contraceptive use has largely focused on a single spouse’s report of family planning discussion with their partner, rather than communication reported by both spouses at the couple level.^25,29–31,34^

Few studies have been conducted using responses from both members of a couple about communication and contraception. A qualitative study conducted in Malawi, based on an evaluation of an intervention exploring the effect of male involvement in family planning, demonstrated that improved couple communication led to increased shared decision-making about family planning and increased use of contraception.^35^ Two studies in Kenya reached different conclusions about the association between couple-level communication and contraceptive use.^27,36^ One found that joint couple reporting of communication about family planning was positively associated with contraceptive use in the urban Kenya context.^27^ However, the other study, based on a nationally representative sample, found no significant relationship between a couple discussion of family planning and actual contraceptive use, even after controlling for couple and individual-level factors.^36^ To our knowledge, this is the first couple-level research conducted about couple communication and contraceptive use in Senegal.

Notably, couple-level research in the Senegalese context is challenging because of the high percentage of polygynous unions: 17% of men and 32% of women are in polygynous unions.^37^ Polygyny is associated with a desire for larger families, along with less communication between partners about family planning and reproductive health, lower contraceptive use rates, and more male extramarital sexual activity.^38–40^ In Niger and Tanzania, women in polygynous unions were less likely to use contraception, even if rates of contraception approval of women in polygynous unions were similar to women in monogamous unions.^39^

In this analysis of couples from urban Senegal, we focused on couple communication about family planning, measured at the couple level, and its influence on current contraception use and future intention to use contraception among non-users. In addition, this research extends the existing literature about couple-level communication, as we used data from a context where a substantial percentage of marriages are polygynous unions, which potentially affects the relationship between couple communication and contraceptive use.

## Methods

### Sample and data source

We conducted secondary data analysis using the cross-sectional baseline individual-level data from the Measurement, Learning, and Evaluation (MLE) Project for the Initiative Sénégalaise de Santé Urbaine (ISSU) originally collected in six urban areas (Dakar, Pikine, Guédiawaye, Mbao, Mbour, and Kaolack) in Senegal in 2011.^41^ The initial primary data collection was done by the MLE Project using a two-stage sampling design. First, a sample of clusters was randomly selected for each of the six cities based on a probability proportional to population. Second, 21 households were randomly chosen in each cluster, creating a sample of about 5500 households. All women who had spent the previous night in the selected households and were aged 15–49 years were eligible to participate in the survey, resulting in a sample of 9614 women. For budgetary reasons, men were only surveyed in four of the cities (Dakar, Pikine, Guédiawaye, and Mbao) and in half of the selected women’s households in those cities.

**Figure 1.**
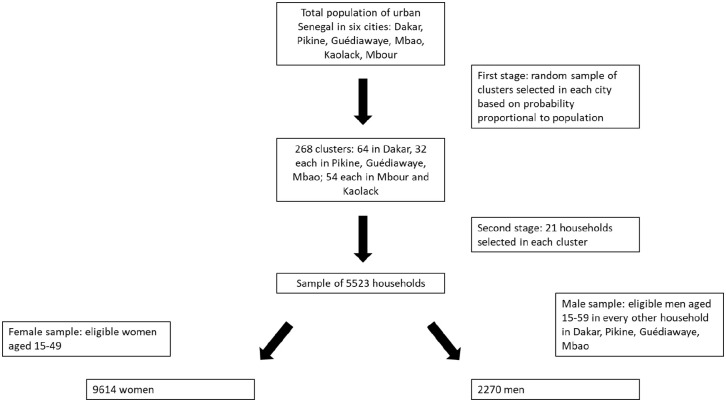
MLE Senegal baseline sampling design.^41,42^

All men who had spent the previous night in the sample of households and were aged 15–59 years were eligible to be interviewed, resulting in a sample of 2270 men.^42^ The sampling strategy of the primary data collection is illustrated in Figure 1 and described in greater detail elsewhere.^43^ All individuals surveyed provided written consent and were interviewed by a trained, same-sex interviewer using a pencil-and-paper survey.

Because our analysis was done at the couple level, our sample size was limited based on the smaller number of cities and households where men were interviewed. Our sample size was further limited to married men (*n* = 835). Only married men and their spouses, if interviewed, were included in our couple sample.

**Figure 2.**
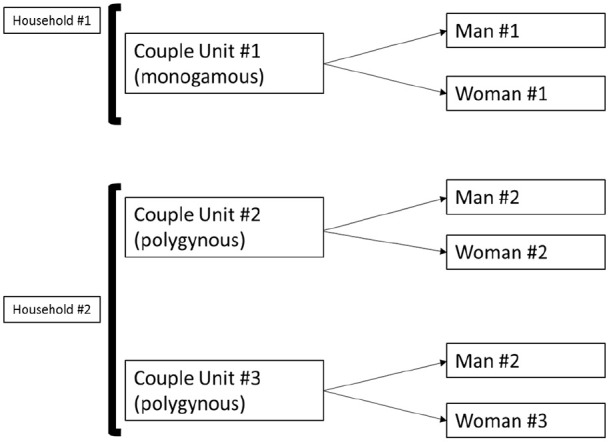
A schematic illustration of creation of couple units.

We constructed couple units within households by identifying heads of household and the spouse(s) of the head of household who had completed interviews (guests or other household members were therefore not included in couple units). We created a unique couple identifier for each couple unit. In polygynous households, we created a couple identifier for the husband and each of his wives, allowing each polygynous household to have more than one couple identifier. The final analysis sample contained 349 couple units, which comprised 332 men and 349 women. A schematic illustration of couple units in both monogamous and polygynous unions is shown in Figure 2.

### Key variables and measures

#### Dependent variables

We had two outcome variables. The primary outcome was woman’s reported current use of contraception. The secondary outcome was woman’s intention to use contraception in the future, asked only to those women not currently using any method (modern or traditional). We defined contraception as any method of contraception or family planning, both modern and traditional methods, including pills, injection, intrauterine devices, implants, male and female condoms, sterilization (both male and female), lactational amenorrhea, fertility awareness (the “rhythm method”), emergency contraception, spermicide, and withdrawal. We include all methods of contraception in our analysis to determine if there is a relationship between couple communication and contraception use, regardless of method.

The primary model (“current use model”) used reported current contraceptive use as the dependent variable, which we define as the woman’s reported use. Woman’s reported use is expected to more accurately represent the *couple*’*s* use of contraception (rather than her husband’s report) due to men possibly reporting use with another wife or an extramarital partner with whom they may have different contraceptive practices, and that women may use contraception clandestinely.^44,45^ For the future intention to use model, we similarly used the woman’s reported intention to use contraception in the next 12 months among contraception non-users. We note that responses for future intention to use were missing for 10 women who were not current contraceptive users; the missing data account for less than 5% of the sample and we treat the missing responses as missing at random and ignorable. Therefore, we conducted analyses for the future intention to use model using complete case analysis, dropping the 10 couples with missing responses.

#### Explanatory variable

The key explanatory variable was spousal communication about family planning, which we defined as a three-level categorical variable. Men and women were asked if they had ever communicated with their spouse about family planning. Based on this information, we defined three levels of couple communication: (1) neither spouse reported communication with his or her partner about family planning; (2) only one spouse reported communication with his or her partner about family planning; or (3) both spouses reported communication with his or her partner about family planning. It is important to note that in polygynous unions, we were unable to determine if the husband’s report refers to communication with all spouses or with only one or some spouses.

#### Covariates

We included several individual-, couple-, and household-level covariates in our models, based on prior research of factors associated with contraceptive use, such as: education, household wealth, ideal number of children, age, number of living children, employment, and polygyny.^12,14–17,20^ The models controlled for fertility preferences at the couple level using reported ideal number of children. We constructed this as a couple-level categorical variable with three levels: (1) the husband’s ideal number of children was higher than the wife’s; (2) the wife’s ideal number of children was higher than the husband’s; and (3) the husband and wife agreed on the ideal number of children. However, 88 women and 147 men in our sample responded that their ideal number of children was “up to God.” For these non-numerical responses, we imputed the median ideal number of children for each sex (four children for women and five children for men). We imputed the medians rather than the means because the distributions for these responses were non-normal and the median represented a better measure of central tendency. As a sensitivity analysis, we ran a model excluding these non-numerical responses (see Supplemental Table 1).

We also constructed a couple-level variable for couple age difference. In previous studies, greater age difference between spouses was associated with less couple communication,^39,46^ so we constructed age as a relational couple-level variable, rather than using individual-level ages. For couple age, there were four categories: (1) the spouses were the same age or the wife was older; (2) the husband was fewer than 5 years older than the wife; (3) the husband was at least 5 but fewer than 10 years older than his wife; or (4) the husband was 10 or more years older than his wife. We also controlled for the wife’s age at the time of survey.

Education was constructed as a six-level categorical variable, also at the couple level: (1) both spouses had no formal education; (2) both had only primary education; (3) the wife had no education and the husband had at least primary education; (4) the wife had a higher level of education than the husband; (5) both partners had at least primary education but the husband had a higher education level than his wife; or (6) both partners had the same level of education that was at least secondary education.

The models controlled for other individual and household characteristics, including men’s and women’s employment in the previous 12 months (defined as working for cash or payment in kind); woman’s reported number of living children; a binary indicator of polygyny as reported by the husband; and household wealth. Because of the overwhelming homogeneity of religion in the sample (95% Muslim) and the small number of non-Muslim individuals in the sample, we did not control for religion in the analyses.

In the sample, 77 of the 332 men reported that they were in polygynous unions. It is important to note, however, that in the analysis sample of couple units, there were only 16 households with polygynous couple units representing 33 separate couples (one household had three wives and therefore had three couple units) and 16 individual men. This could be the result of wives not being eligible for interview, living elsewhere, or simply not being interviewed or not completing the interview. For analysis in the models, we controlled for polygyny based on the husband’s responses about the type of union (binary yes/no if they report being in polygynous unions), rather than if all of their spouses were represented in the sample. Otherwise, polygyny would be artificially under-represented and not appropriately controlled for in the sample. As an additional sensitivity analysis, we ran a model using woman’s self-reported use of any contraception using only monogamous couples (as reported by the husband) (*n* = 255 couples, 77% of sample) to determine if associations were similar compared to the whole sample that included polygynous couples (see Supplemental Table 2).

### Analysis

We ran multivariable logit models with the individual-, couple-, and household-level covariates described above for both current contraceptive use and the future intended use (among women who reported current non-use). The unit of analysis was the couple. Although clustering was only a concern in less than 5% of the sample, we ran models with standard errors clustered at the household level to account for correlation between polygynous couples in the same household. We did not observe any changes in significance level compared to models with unclustered standard errors, and differences in standard errors for all variables were very small (within two thousandths between models for all covariates); we therefore report unclustered robust standard errors. The current use model tested the association of a couple discussing family planning with the woman’s reported current use of contraception. The future intention to use contraception model tested the association of a couple discussing family planning with woman’s intended use of contraception, among women who report current non-use.

As a supplemental analysis, we ran a model using women’s self-reported current use of *modern* contraception rather than any form of contraception as the outcome variable. We defined modern contraception according to the Demographic and Health Surveys and excluded natural and traditional methods.^16,37^ We cannot report the association with future intention to use modern methods, as women were not specifically surveyed about future intended use of *modern* contraception (see Supplemental Table 3). All statistical analyses were conducted using STATA 16.1 (College Station, Texas, USA).

## Results

### Demographics

The sample included 332 husbands and 349 wives, for a total of 349 couple units. In total, 40% of husbands and 34% of wives reported any contraceptive use, and 32% of husbands and 30% of wives reported using modern methods. Among those who reported no contraception use, 31% of husbands and 26% of wives reported they intended to use contraception in the next 12 months. At the individual level, husbands were about 10 years older than wives on average (43 years compared to 33 years). About 44% of wives and 37% of husbands did not have any formal education. Nearly 95% of husbands reported employment in the previous year versus 59% of wives. Husbands had, on average, 4.5 living children, while wives reported 3.2 living children. Selected individual-level demographic characteristics are presented in Table 1.

**Table 1.** Selected individual-level characteristics of husbands (*n* = 332) and wives (*n* = 349) in urban Senegal sample, 2011.

No. of observations	Husbands %	Wives %
	332	349
Discussed family planning with partner	52.1	59.5
Ideal number of children (SD)	5.87 (3.11)	4.57 (1.49)
City		
Dakar	34.9	34.4
Guédiawaye	22.6	23.2
Pikine	21.1	20.9
Mbao	21.4	21.5
Age (SD)	42.80 (8.14)	32.59 (7.80)
In polygynous union	23.5	26.9
Education		
No education	36.6	44.4
Primary	24.2	35.0
Secondary	29.3	15.5
Higher than secondary	10.0	5.2
Wealth quintiles		
Poorest	28.9	27.8
Second	30.7	30.9
Middle	15.7	15.2
Fourth	14.2	14.9
Richest	10.5	11.2
Employed previous 12 months	94.6	58.9
No. of living children (SD)	4.48 (3.45)	3.20 (2.20)
Any current contraceptive use	39.8	33.5
Contraception method type (self or partner)^a^		
Pills	30.3	33.3
Injectable	20.5	32.5
Implant	6.8	5.1
Intrauterine device	5.3	5.1
Condom	18.9	10.3
Spermicide	0	0.9
Sterilization	0	2.6
Natural methods	15.2	8.5
Lactational amenorrhea	1.5	0.9
Other	1.5	0.9
Intention to use contraception in future^b^	31.0	27.5

a Among 132 husbands and 117 wives who report current use of contraception.

b Among 200 husbands and 232 wives who report no current use of contraception.

*Note*: Table contains weighted demographic percentages adjusted by city weight to account for survey design.

At the couple level, only 3.7% of spouses were the same age (or the wife was older). In more than half (52.1%) of the couples, the husband was 10 or more years older than his wife. Neither spouse had any formal education in 22.3% of the couples, and in 9.2% of couples, both spouses had only primary education. In 63.6% of couples, the husband’s ideal number of children was higher than the wife’s ideal number. All couple-level variables are reported in Table 2 (*n* = 349 couples).

**Table 2.** Couple-level characteristics of full couple sample (*n* = 349) from urban Senegal, 2011.

Characteristic	Full couple sample %
Couple report of discussing family planning	
Neither report	27.2
One spouse reports	34.4
Both report	38.4
Couple age	
Spouses are the same age or wife older	3.7
Husband is within 4 years of wife	16.3
Husband more than 5 but fewer than 10 years older than wife	27.8
Husband more than 10 years older than wife	52.1
Couple education	
Both no education	22.3
Both primary education	9.2
Husband has any level of education and wife has no education	22.1
Wife has higher level of education than husband	20.3
Both partners have at least primary education but husband has higher education level than wife	18.1
Both partners have same level of education and higher than primary education	8.0
Ideal number of children	
Husband’s ideal number is larger than wife’s ideal number	63.6
Wife’s ideal number is larger than husband’s ideal number	20.1
Equal husband and wife ideal number	16.3

### Explanatory variables: couple communication

Couple communication about family planning varied such that 52.1% of men and 59.5% of women reported discussing family planning with their spouses. At a couple level, in 38.4% of couples, communication about family planning was reported by both spouses; in 34.4% of couples, communication was reported by one spouse; and in 27.2% of couples, neither spouse reported communication about family planning. In couples where only one spouse reported communication, 61% were wives and 39% were husbands; a sensitivity test using a four-level communication variable showed no significant difference in likelihood to use contraception between a husband and wife reporting discussing family planning and the other not reporting discussion.

### Multivariable analyses: reported use of contraception

Table 3 presents the average marginal effects of reported couple communication about family planning and its association with the wife’s reported current use of contraception or future intention to use contraception. In the current use model, couples in which one spouse reported discussing family planning were associated with a 24.6% point increase in the probability of the wife’s reported use of contraception (*p* < 0.01) compared to couples where neither spouse reported discussion. Couples in which both spouses reported discussing family planning were associated with a 55.7% point increase in the likelihood of the wife’s reported use of contraception (*p* < 0.01) compared to couples where neither spouse reported family planning discussions.

**Table 3.** Average marginal effects of selected variables’ association with use of any contraception (among all couples) and future intention to use contraception (among couples not currently using contraception).

Individual or couple variable	All couples (*n* = 349)	Couples not currently using contraception (*n* = 222)
	Average marginal effect (Delta-method SE)	Average marginal effect (Delta-method SE)
Couple report discussing family planning		
Neither report discussing	referent	referent
One spouse reports discussing	0.246** (0.044)	0.149* (0.058)
Both report discussing	0.557** (0.046)	0.380** (0.080)
Couple age		
Spouses are the same age or wife older	referent	referent
Husband is within 4 years of wife	0.206* (0.089)	0.234* (0.113)
Husband at least 5 and fewer than 10 years older than wife	0.126 (0.085)	0.162 (0.093)
Husband more than 10 years older than wife	0.245** (0.088)	0.168 (0.097)
Couple ideal number of children		
Equal husband and wife ideal number	referent	referent
Husband’s ideal number is larger than wife’s ideal number	0.001 (0.056)	0.040 (0.077)
Wife’s ideal number is larger than husband’s ideal number	−0.147 (0.067)	−0.058 (0.087)
Couple education		
Both no education	referent	referent
Both primary education only	0.127 (0.093)	0.154 (0.101)
Husband has at least primary education and wife has no education	0.171** (0.060)	0.009 (0.080)
Wife has higher level of education than husband	0.224** (0.069)	0.046 (0.095)
Both spouses have at least primary education; husband has more education than wife	0.213** (0.079)	0.004 (0.101)
Both spouses same; higher than primary education	0.194* (0.097)	0.187 (0.158)
Wife number of living children (SD)	0.042** (0.015)	0.073** (0.016)
Wife age (SD)	−0.002 (0.004)	−0.020** (0.005)
Polygynous union (husband report)	−0.066 (0.055)	−0.019 (0.067)
Wife employed in previous 12 months	−0.014 (0.045)	0.013 (0.061)
Husband employed in previous 12 months	0.165 (0.135)	−0.146 (0.120)

* *p* < 0.05, **p < 0.01; *Notes*: models also control for household wealth quintile; contraceptive use based on wife’s report; McFadden’s ρ^2^ = 0.304 for all couples model.

In the current use model, some categories of the relational couple age variable were significantly associated with the wife’s reported use of contraception (Table 3). A husband being 10 or more years older than his wife was associated with a 24.5% point increase in the likelihood of the wife’s reported use of contraception, compared to couples who are the same age or the wife is older (*p* < 0.01). The wife’s own age was not significantly associated with the wife’s reported use of contraception.

Discordant couple-level ideal number of children was not associated with a greater likelihood of the wife’s reported contraceptive use, compared to couples who agreed about their ideal number of children. Any couple-level education combination where the wife had at least primary education (except where both spouses had only primary education) was associated with a significant increase in the likelihood of the wife’s reported use compared to couples where both spouses had no formal education (all *p*s < 0.05).

The number of living children, as reported by the wife, was significantly associated with an increase in the likelihood of the wife’s reported use of contraception. Every additional living child was associated with a 4.2% point increase in the likelihood of the wife’s reported use of contraception (*p* < 0.01). Husband-reported polygynous unions were negatively associated with the wife’s reported contraceptive use, but the association was not statistically significant.

### Multivariable analyses: intended future use of contraception

Table 3 also presents the average marginal effects of a couple reporting discussing family planning and its association with the wife’s future intended use of contraception in the next 12 months, among wives who reported non-use of contraception (*n* = 222 wives and couple units). Couples in which one spouse reported discussing family planning were associated with a 14.9% point increase (*p* < 0.05) in the likelihood of the wife’s reported intention to use contraception in the next 12 months, compared to couples in which neither spouse reported discussing family planning. Couples in which both spouses reported discussing family planning were associated with a 38.0% point increase (*p* < 0.01) in the wife’s intention to use contraception in the next 12 months, compared to couples in which neither spouse reported discussing family planning.

The future intention to use model had other notable results among the couple-level and individual covariates. Unlike results in the current use model, couples in which a husband was 10 or more years older than his wife were not significantly associated with the likelihood of the wife’s reported intention to use contraception, compared to couples who were the same age or in which the wife was older. And, in the future intention to use model, the wife’s age was significantly associated with her intention to use contraception. Every 1-year increase in the wife’s age was associated with a 2.0% point decrease in likelihood of her intention to use contraception in the next 12 months (*p* < 0.01). We again observed no significant association between the husband’s reported polygyny status and the wife’s intention to use contraception.

### Supplemental and sensitivity analyses

In the supplemental analysis model using current *modern* contraceptive method use as the outcome (Supplemental Table 3), results were similar to those presented above. A couple jointly reporting discussing family planning was associated with a 48.4% point increase in the probability of the wife’s reported use of modern contraception (*p* < 0.01) compared to couples in which neither spouse reported discussing family planning. Couples in which one spouse reported discussing family planning were associated with a 22.0% point increase in family planning use (*p* < 0.01) compared to couples where neither spouse reported discussing family planning. Using reported current modern contraceptive use as the outcome, rather than any contraceptive use, did not dramatically change the results; couples where both spouses reported discussing family planning were highly significant, and the magnitude of the effect was large for both current modern contraceptive use and any contraceptive use (see Supplemental Table 3).

In the sensitivity analysis excluding non-numerical responses of ideal number of children, we found that results were similar in significance to the full model estimates (see Supplemental Table 1). Finally, in the sensitivity analysis model that used exclusively monogamous couples (*n* = 255, based on husband’s report of polygyny status), both spouses reporting family planning discussion was associated with a 59.5% point increase in the likelihood of reporting use of any contraceptive method (*p* < 0.01). Couples in which only one spouse reported discussing family planning were associated with a 26.8% point increase in likelihood of reporting any contraceptive use (*p* < 0.01). The magnitude of the association between couple discussion of family planning and contraceptive use was especially high in the monogamous couples sample (see Supplemental Table 2).

## Discussion

In this study, we found large, positive, and significant associations between couple discussion of family planning, measured at the couple level, and the wife’s reported current contraceptive use and future intention to use. The magnitude of the effect was approximately twice as large if both spouses reported discussion of family planning in all models, compared to couples where only one spouse reported discussion. As expected, we found several other couple-level and individual covariates significantly associated with contraceptive use and intended use, but we did not find that polygyny was significantly associated with current contraceptive use or future intended use.

Much of the literature to this point has used only one spouse’s recollections, typically the wife’s, of discussion of family planning to represent couple communication.^25,29,31,34^ Our findings highlight the importance of using couple-level variables and couple-level analysis to determine the association of couple communication and contraceptive use. We demonstrated that relying on the report of only one spouse attenuates the association of couple communication and contraceptive use and underscores the importance of shared perceptions of communication with respect to contraceptive use and planned future use. The higher likelihood of use in couples with jointly reported discussion is consistent with findings about couple communication in other geographic regions. For example, a recent study in rural India found that compared to no reported couple communication, both concordant and discordant communication (only one partner reporting communication) were associated with contraceptive use.^47^

We found other noteworthy results in our analysis. Having a mismatched ideal number of children (either spouse wanting more children than the other) within a couple was not significantly associated with an increase in the wife’s reported use, compared to couples who had an equal value for the ideal number of children, in any of the models. This finding was somewhat unexpected; in a recent study in Ethiopia, researchers found that a husband’s desire for additional children was positively and significantly associated with contraceptive use in situations when there was discordance and the wife did not want more children.^25^ Our findings could be partially the result of imputing sex subsample medians for fatalistic (“up to God”) responses about the ideal number of children, thus limiting variation both within husbands and wives subsamples and between the sexes. This is likely especially true for husbands, where we imputed the median for 44% of the sample. Preferably, all responses to the ideal number of children question would be numerical as this would enhance the ability to draw more robust conclusions; however, forcing only numerical responses may be inappropriate or unfeasible.

In every model, the number of living children the wife has was positively and significantly associated with likelihood of contraceptive use and intention to use in the future, even after controlling for the wife’s age. Older women are more likely to have higher parity, having been in their reproductive years for longer, and increasing age has been found to be positively associated with increased likelihood of contraceptive use (up to approximately 35 years of age).^48,49^ These findings likely reflect a tendency to avoid or delay having additional children as women approach their ideal family size and are consistent with findings in other settings.^33,49^

The current use model demonstrated a positive association of wife’s education level within a couple and contraceptive use, which was anticipated and consistent with the existing body of literature.^50–53^ In couples in which the wife had at least a primary level of education (except in couples where *both* spouses only had a primary level of education), couple-level variable combinations of education level were positively and significantly associated with reported contraceptive use.

This research did not find that polygyny was significantly associated with use of contraception or with future intention to use contraception among non-users, after controlling for other individual, couple, and household characteristics. It is tempting to consider this finding surprising, as polygyny has been found to be associated strongly with pronatalism and less willingness to use contraception.^34,39^ However, some research has noted that there are overarching sociocultural values that “transcend marriage types,” and that any differences observed between marriage types could be attributable to the localized influence of cultural and community factors where polygyny exists; women in monogamous and polygynous unions may be equally influenced by predominant pronatalist norms of their community.^53–55^ For example, a quantitative study in Ethiopia found no link between contraceptive use and polygyny, and the researchers attributed this finding to the comparatively stronger influences of local culture, including community factors, such as religion and access to family planning services.^53^ Our findings lend support to the contention that polygyny’s influence in family planning decision-making is limited compared to the cultural and community influences in the context in which polygyny occurs.^54^

There are several limitations of this research. The measure of discussion of family planning potentially suffers from recall bias and is not randomly assigned, so it could be picking up an omitted variable. It is possible that if individuals are more likely to remember a discussion about family planning, the more impactful it was, or the more it may change family planning behavior. Because of this limitation, we present associations rather than causal estimates. Unique to contexts with polygynous couples, we were also unable to determine if the husband’s report of discussion of family planning was applicable to all spouses or to only one or some spouses. We treated a husband’s report of discussing family planning as applicable to all of his wives, which may have over-represented these discussions. However, by including a measure that considers both spouses reporting, this potential measurement error is likely limited. There is also limited generalizability for these findings, as the sample is comprised solely of couples from urban Senegal. Since our findings are consistent with results from similar research in urban Kenya,^27^ there may be some evidence for generalizability to other urban regions in sub-Saharan Africa, though more research is needed in other contexts. In addition, though our goal was to maximize the number of couples analyzed, because of constraints of the primary data, the sample size of couples was relatively small. Due to the limited sample size, we report associations between variables and do not make causal inferences. Future couple-level research should identify and survey both spouses of couples at the time of data collection to increase the sample size and consider expanding the sample to include unmarried couples in other contexts. Having a larger sample size of couples would allow for additional analyses, for example, whether spousal communication is associated with use of specific contraceptive methods. However, our study is the first to our knowledge that uses couple-level analysis in the Senegalese context to determine the association between couple communication and contraceptive use.

Interventions aimed at increasing contraceptive prevalence rates may be enhanced by providing couples with tools for starting conversations about family planning or providing support and information about communicating with a spouse, rather than simply providing information about contraception to one or both spouses separately. Studies in India have noted success with community-based interventions and capacity building of health care providers to encourage spousal discussion of family planning, along with leveraging local media, engaging community leaders in messaging, or involving other family members (e.g. mothers in law) to promote communication about family planning and increase uptake of contraception.^56,57^ And research in Ethiopia found that household-level family planning education in conjunction with community gatherings increased spousal communication about family planning.^58^

## Conclusion

These findings demonstrate a clear positive association between couple communication about family planning and contraceptive use, measured at the couple level, in the Senegalese context. They underscore the importance of measuring communication at the couple level: both spouses reporting discussions of family planning was associated with approximately twice the likelihood of contraceptive use than if a single spouse reported a family planning discussion, compared to couples in which neither spouse reported discussion. The findings were consistent across all tested models. If both spouses recall the conversation, it may be more likely that discussion actually shapes contraceptive behavior, as these results indicate, and suggests that family planning decisions are more often made jointly in these couples, a factor associated with higher rates of contraceptive use. This research additionally showed a positive association between couple communication about family planning and future intention to use contraception among women who reported non-use. Family planning programs could encourage couple communication and include male partners to positively impact uptake of contraception and planned future use of contraception among couples in urban Senegal.

## Supplemental Material

sj-pdf-1-smo-10.1177_20503121211023378 – Supplemental material for Couple communication and contraception use in urban Senegal Click here for additional data file. Supplemental material, sj-pdf-1-smo-10.1177_20503121211023378 for Couple communication and contraception use in urban Senegal by Brigid K Grabert, Ilene S Speizer, Marisa Elena Domino, Leah Frerichs, Amy Corneli and Bruce J Fried in SAGE Open Medicine

sj-pdf-2-smo-10.1177_20503121211023378 – Supplemental material for Couple communication and contraception use in urban Senegal Click here for additional data file. Supplemental material, sj-pdf-2-smo-10.1177_20503121211023378 for Couple communication and contraception use in urban Senegal by Brigid K Grabert, Ilene S Speizer, Marisa Elena Domino, Leah Frerichs, Amy Corneli and Bruce J Fried in SAGE Open Medicine

sj-pdf-3-smo-10.1177_20503121211023378 – Supplemental material for Couple communication and contraception use in urban Senegal Click here for additional data file. Supplemental material, sj-pdf-3-smo-10.1177_20503121211023378 for Couple communication and contraception use in urban Senegal by Brigid K Grabert, Ilene S Speizer, Marisa Elena Domino, Leah Frerichs, Amy Corneli and Bruce J Fried in SAGE Open Medicine
