# Supplementary material for: Couple communication and contraception use in urban Senegal
Source: SAGE Open Med. 2021 Jun 4;9:20503121211023378. doi: 10.1177/20503121211023378 (PMC8182225; doi:10.1177/20503121211023378)
Supplement: sj-pdf-3-smo-10.1177_20503121211023378 – Supplemental material for Couple communication and contraception use in urban Senegal [file sj-pdf-3-smo-10.1177_20503121211023378.pdf]

Supplemental Table 3: Average marginal effects of selected variables' association with use of *modern* contraception in couples

	**Couples (n=349)**
**Individual or Couple Variable**	**Average marginal effect (Delta-method std. error)**
Couple report discussing family planning	
Neither report discussing	referent
One spouse reports discussing	0.220** (0.044)
Both report discussing	0.484** (0.048)
Couple age	
Spouses are the same age or wife older	referent
Husband is within 4 years of wife	0.173 (0.094)
Husband at least 5 and fewer than 10 years older than wife	0.077 (0.087)
Husband 10 or more years older than wife	0.200* (0.090)
Couple ideal number of children	
Equal husband and wife ideal number	referent
Husband's ideal number is larger than wife's ideal number	0.023 (0.058)
Wife's ideal number is larger than husband's ideal number	0.005 (0.070)
Couple education	
Both no education	referent
Both primary education only	0.046 (0.089)
Husband has at least primary education and wife has no education	0.119* (0.061)
Wife has higher level of education than husband	0.172* (0.069)
Both partners have at least primary education; husband has more education than wife	0.201* (0.079)
Both partners same; higher than primary education	0.194 (0.103)
Wife number of living children (std dev)	0.042** (0.014)
Wife age (std dev)	-0.005 (0.005)
Polygynous union (husband report)	-0.049 (0.057)
Wife employed in previous 12 months	-0.000 (0.047)
Husband employed in previous 12 months	0.113 (0.133)

* $p<0.05$, ** $p<0.01$; *Notes*: models also control for household wealth quintile; contraceptive use based on wife's report
